# P-gp expression inhibition mediates placental glucocorticoid barrier opening and fetal weight loss

**DOI:** 10.1186/s12916-021-02173-4

**Published:** 2021-12-08

**Authors:** Caiyun Ge, Dan Xu, Pengxia Yu, Man Fang, Juanjuan Guo, Dan Xu, Yuan Qiao, Sijia Chen, Yuanzhen Zhang, Hui Wang

**Affiliations:** 1 Department of Obstetrics and Gynaecology, Zhongnan Hospital of Wuhan University, 169 Donghu Road, Wuchang District, Wuhan, 430071 China; 2 Department of Pharmacology, Basic Medical School of Wuhan University, 185 Donghu Road, Wuchang District, Wuhan, 430071 China; 3 Hubei Provincial Key Laboratory of Developmentally Originated Diseases, 185 Donghu Road, Wuchang District, Wuhan, 430071 China

**Keywords:** Intrauterine growth retardation, P-glycoprotein, Placental glucocorticoid barrier, Prenatal caffeine exposure, Histone acetylation

## Abstract

**Background:**

Prenatal adverse environments can cause fetal intrauterine growth retardation (IUGR) and higher susceptibility to multiple diseases after birth, related to multi-organ development programming changes mediated by intrauterine overexposure to maternal glucocorticoids. As a glucocorticoid barrier, P-glycoprotein (P-gp) is highly expressed in placental syncytiotrophoblasts; however, the effect of P-gp on the occurrence of IUGR remains unclear.

**Methods:**

Human placenta and fetal cord blood samples of IUGR fetuses were collected, and the related indexes were detected. Pregnant Wistar rats were administered with 30 mg/kg·d (low dose) and 120 mg/kg·d (high dose) caffeine from gestational day (GD) 9 to 20 to construct the rat IUGR model. Pregnant mice were administered with caffeine (120 mg/kg·d) separately or combined with sodium ferulate (50 mg/kg·d) from gestational day GD 9 to 18 to confirm the intervention target on fetal weight loss caused by prenatal caffeine exposure (PCE). The fetal serum/placental corticosterone level, placental P-gp expression, and related indicator changes were analyzed. In vitro, primary human trophoblasts and BeWo cells were used to confirm the effect of caffeine on P-gp and its mechanism.

**Results:**

The placental P-gp expression was significantly reduced, but the umbilical cord blood cortisol level was increased in clinical samples of the IUGR neonates, which were positively and negatively correlated with the neonatal birth weight, respectively. Meanwhile, in the PCE-induced IUGR rat model, the placental P-gp expression of IUGR rats was decreased while the corticosterone levels of the placentas/fetal blood were increased, which were positively and negatively correlated with the decreased placental/fetal weights, respectively. Combined with the PCE-induced IUGR rat model, in vitro caffeine-treated placental trophoblasts, we confirmed that caffeine decreased the histone acetylation and expression of P-gp via RYR/JNK/YB-1/P300 pathway, which inhibited placental and fetal development. We further demonstrated that P-gp inducer sodium ferulate could reverse the inhibitory effect of caffeine on the fetal body/placental weight. Finally, clinical specimens and other animal models of IUGR also confirmed that the JNK/YB-1 pathway is a co-regulatory mechanism of P-gp expression inhibition, among which the expression of YB-1 is the most stable. Therefore, we proposed that YB-1 could be used as the potential early warning target for the opening of the placental glucocorticoid barrier, the occurrence of IUGR, and the susceptibility of a variety of diseases.

**Conclusions:**

This study, for the first time, clarified the critical role and epigenetic regulation mechanism of P-gp in mediating the opening mechanism of the placental glucocorticoid barrier, providing a novel idea for exploring the early warning, prevention, and treatment strategies of IUGR.

**Supplementary Information:**

The online version contains supplementary material available at 10.1186/s12916-021-02173-4.

## Background

Intrauterine growth retardation (IUGR) refers to a fetus that grew slowly in the uterus and failed to achieve the expected weight for gestational age [1]. IUGR usually results in growth retardation, multiple organ dysfunction, and low birth weight, which can cause fetal distress, neonatal asphyxia, and even perinatal death [2, 3]. The epidemiological survey has shown that the incidence of IUGR was about 3–9% in socioeconomically developed areas, while it was as high as 30% in economically underdeveloped areas [4]. Children with IUGR, especially those who had catch-up growth during early life, have a higher risk of susceptibility to various diseases, including metabolic diseases (MS), type 2 diabetes, cardiovascular diseases, and mental diseases in later life [5–8]. Besides, animal studies have revealed that adverse environments during pregnancy (such as xenobiotics exposure and dietary restrictions, etc.) can increase the incidence of IUGR and the susceptibility to many chronic diseases after birth [9–11]. At present, there are still many difficulties in the prevention and treatment of IUGR, mainly related to the unclear mechanism of occurrence and target of intervention.

Glucocorticoids play an essential role in maintaining pregnancy and regulating embryonic tissue morphology and function maturity [12]. However, overexposure to glucocorticoids caused by various prenatal factors may be a trigger for adverse developmental outcomes (e.g., IUGR), affecting fetal developmental programming and leading to susceptibility to a variety of fetal-originated diseases in adults [13–15]. Maternal glucocorticoid levels in the fetus are mainly regulated by the placental glucocorticoid barrier, including 11β-hydroxysteroid dehydrogenase type 2 (11β-HSD2) and P-glycoprotein (P-gp). The 11β-HSD2 can reduce glucocorticoids from entering fetal circulation by inactivating glucocorticoids [15]. In our previous studies, through a series of animal experiments, we have established stable IUGR offspring rat models caused by prenatal xenobiotics exposure such as caffeine, nicotine, and ethanol and confirmed that prenatal xenobiotics exposure induced-IUGR was associated with a low expression level of placental 11β-HSD2 [10, 16, 17]. P-gp is one of the most abundantly expressed proteins of the ATP binding cassette efflux transporter family in the placentas. P-gp in the placental trophoblasts can use ATP to pump glucocorticoids back to the maternal circulation [18]. However, the effect of P-gp as a placental glucocorticoid barrier on the occurrence of IUGR is unclear.

Clinical and animal studies have found that adverse environments during pregnancy can cause IUGR. Caffeine is the most widely consumed psychoactive drug in the world. According to statistics, 82% of pregnant women have been reported to consume caffeine daily in the USA [19] and 91% in France [20]. Epidemiological investigations have proved that prenatal caffeine exposure (PCE) can cause toxic effects of reproduction and fetal development, such as the increased risk of congenital malformations, premature delivery, spontaneous abortion, IUGR, and susceptibility to chronic diseases [21, 22]. Our animal experiments also demonstrated that PCE could not only increase maternal glucocorticoid levels but also cause fetal overexposure to glucocorticoid by inhibiting placental 11β-HSD2, leading to the occurrence of IUGR and susceptibility to multiple chronic diseases in adult offspring [16, 23–26]. However, whether caffeine causes IUGR by affecting placental P-gp has not been reported yet. Epigenetic changes are critical to gene regulation in the developing placenta. As an essential transporter of syncytiotrophoblast, the epigenetic regulation mechanism of placental P-gp is rarely reported. It has been suggested [27, 28] that the JNK pathway and Y-box binding protein-1 (YB-1) may be involved in P-gp regulation. It is not known whether the JNK pathway, YB-1, and epigenetic modifications are involved in the regulation of placental P-gp by caffeine.

Here, using clinical specimens and PCE-induced IUGR rat model, we first determined the glucocorticoid barrier function of placental P-gp in the IUGR neonates and fetal rats by analyzing the correlations among the placental P-gp expression levels, placental/fetal glucocorticoid levels, and placental/fetal body weights. Furthermore, by combining in vivo experiments of caffeine, the clinical IUGR samples, and rat IUGR models, we will clarify the epigenetic mechanism of P-gp expression changes in placental trophoblast cells. Next, P-gp inducer sodium ferulate will be used to confirm the intervention target on fetal weight loss caused by PCE. Finally, we will confirm the potential early warning targets of IUGR occurrence and multiple disease susceptibility using clinical specimens. This study will illuminate the underlying mechanism of IUGR from the perspective of placental P-gp and provide a novel idea for analyzing the placental origin of adult diseases, as well as the exploration of early warning targets and potential intervention strategies.

## Methods

### Chemicals and reagents

Caffeine (C0750) and cortisol (C46329) and DNase I (DN25) were obtained from Sigma-Aldrich Co., Ltd. (St Louis, MO, USA). Rat corticosterone ELISA kits (RE52211) and cortisol ELISA kits (RE52061) were purchased from IBL International (Germany). Dantrolene (B6329), SP600125 (A4604), and Rhodamine 123 (Rho 123, C3140) were purchased from APExBIO Technology LLC (Houston, MA, USA). Trypsin (9002-07-7) and lipofectamine 3000 (L3000015) were purchased from Gibco Co. (Detroit, MI, USA). YB-1 pcDNA3.1( + ) plasmid was constructed by GenePharma Co., Ltd. (Shanghai, China). TRIzol (15596018) reagent was purchased from Invitrogen Co. (Carlsbad, CA, USA). The quantitative real-time PCR (qRT-PCR) reverse transcription (R312) and SYBR qPCR master mix kits (Q711) were purchased from Vazyme Biotech Co., Ltd. (Nanjing, China). The nuclear and cytoplasmic protein preparation kit (P1200) and BCA assay kit (P1511) were purchased from Applygen Co., Ltd. (Beijing, China). Primary antibodies anti-P-gp (ab170904), anti-YB-1 (ab76149), anti-P300 (ab14984), anti-acetyl H3K9 (ab10812), anti-acetyl H3K14 (ab52946), and IgG (ab172730) antibody were purchased from Abcam Technology Co., Ltd. (Cambridge, UK). The anti-JNK (A4867), anti-p-JNK (AP0631), anti-H3 (A2348), and anti-GAPDH (A10868) antibodies were purchased from ABclonal Technology Co., Ltd. (Wuhan, China). The enhanced chemiluminescence kit (ECL, 32209) was obtained from Pierce Biotechnology Inc. (Rockford, IL, USA). Chromatin immunoprecipitation (ChIP) assay kit (17-295) was purchased from Millipore Co., Ltd. (Billerica, MA, USA). DNA purification kit (DP214) was purchased from Tiangen Biotech Co., Ltd. (Beijing, China). The other reagents for experiments were of analytical grade.

### Human placental tissue collection

The placentas from IUGR neonates were collected after obtaining informed consent from pregnant women who underwent a cesarean section or natural delivery at the Division of Gynecology and Obstetrics of Zhongnan Hospital, Wuhan University, between June 2017 and June 2018. It was approved by the Medical Ethical Committee of Zhongnan Hospital of Wuhan University (approval number 201606). IUGR was diagnosed according to an intrauterine condition when a baby’s weight is below the 10th centile for gestational age [29]. The following criteria were required for the inclusion: singleton pregnancy, gestational age within 28–40 weeks, and the weight of the fetus in the control group is adapted to the gestational age, while the weight of the fetus in the IUGR group is lower than the 10th percentile of the gestational age. The fetus with any congenital genetic diseases, fetal structural, genetic, or chromosomal abnormalities was excluded. Pregnancy complications, including pre-eclampsia and gestational diabetes, are also adverse environments during pregnancy since they can cause maternal stress and HPA axis activation [30, 31], which is consistent with the research goal that we want to study the adverse environments during pregnancy leading to fetal weight loss. Therefore, women with pregnancy complications were not excluded from the IUGR group. Thirty control and 26 IUGR neonatal placentas, 89.3% of cesarean section, and 10.7% (3/30 in the control group, 3/26 in the IUGR group) of natural delivery were finally recruited. The samples of the control group were collected from healthy women, and the samples of the IUGR group were collected from healthy women or women with pregnancy complications (15 cases of pre-eclampsia, 6 cases of gestational diabetes, 2 cases of nuchal cord, and 1 case of oligohydramnios). The placental tissues and umbilical cord blood were collected immediately after delivery. The placental tissue was dissected at the middle zone after the amniotic membranes, decidua, and connective tissues had been removed. Partial placental tissue was fixed with 10% paraformaldehyde, and partial tissue was immediately snap-frozen in liquid nitrogen and stored at -80°C after being washed thoroughly with saline. Fetal cord serum was isolated from umbilical cord blood by centrifugation.

### Animals and treatment

Specific pathogen-free Wistar rats and C57BL/6 mice were obtained from the Experimental Center of Hubei Medical Scientific Academy (No. 2017-0018, certification number: 42000600002258, Hubei, China). The animal protocol of rats was reviewed and approved by the Ethics Committee of Wuhan University School of Medicine (No. 14016), and the animal protocol of mice was reviewed and approved by the Institutional Animal Care and Use Committee (IACUC) of Wuhan University Center for Animal Experiment (No. WP20210003). All the protocols were conformed to the National Institutes of Health guide for the care and use of laboratory animals.

Details of rat feeding and mating were described as before [32]. Upon confirmation of mating by the appearance of sperm in a vaginal smear, the day was defined as the gestational day (GD) 0. From GD9 to GD20, the rats in PCE groups were intragastrical administered 30 and 120 mg/kg·d caffeine (dissolved in distilled water) according to the PCE(L) (low dose) and PCE(H) (high dose) groups, the prenatal ethanol exposure (PEE) group was given 4 g/kg·d ethanol (dissolved in distilled water) by gavage administration, while the control group was given the same volume of distilled water. Pregnant rats were injected subcutaneously with 0.8 mg/kg·d dexamethasone (dissolved in saline) for the prenatal dexamethasone exposure (PDE) group or saline for the control group. On GD20, pregnant rats were sacrificed after being anesthetized at 8 am. The sex of the fetal rats was judged by the anogenital distance and then was ensured by the anatomy of the testicles or ovaries. The fetal blood of the same sex in each litter was combined into a single specimen. Maternal and fetal serum was isolated from the blood by centrifugation (3500 rpm) for 15 min. The placental tissue from three different fetal rats from each litter was randomly collected, pooled, and counted as one sample according to gender for further RNA and protein extraction according to methods previously described [33]. Partial placental tissue was fixed with 4% paraformaldehyde for immunohistochemistry assay. While the other samples were frozen immediately in liquid nitrogen, followed by storage at -80°C for subsequent examination.

$$ {\displaystyle \begin{array}{c}\mathrm{IUGR}\ \mathrm{rate}\ \mathrm{per}\ \mathrm{litter}\ \left(\%\right)=\left(\mathrm{number}\ \mathrm{of}\ \mathrm{IUGR}\ \mathrm{rat}\ \mathrm{fetuses}\ \mathrm{per}\ \mathrm{litter}/\mathrm{the}\ \mathrm{total}\ \mathrm{number}\ \mathrm{of}\ \mathrm{fetal}\ \mathrm{rats}\ \mathrm{per}\ \mathrm{litter}\right)\times 100\\ {}\mathrm{IUGR}\ \mathrm{rate}\ \mathrm{per}\ \mathrm{group}\ \left(\%\right)=\left(\mathrm{the}\ \mathrm{sum}\ \mathrm{of}\ \mathrm{fetal}\ \mathrm{rat}\ \mathrm{IUGR}\ \mathrm{rate}\ \mathrm{per}\ \mathrm{litter}/\mathrm{each}\ \mathrm{group}\ \mathrm{of}\ \mathrm{litter}\ \mathrm{number}\right)\times 100\end{array}} $$

For the intervention study of mice, the pregnant mice were randomly divided into four groups, namely the control group (control), the caffeine group (caffeine), the sodium ferulate group (sodium ferulate), and the caffeine + sodium ferulate group (caffeine + sodium ferulate). From GD9 to GD18, both caffeine (120 mg/kg·d) and sodium ferulate (50 mg/kg·d) were administered by gavage, while the control group was given the same volume of distilled water. On GD18, pregnant mice were sacrificed after anesthetized. IUGR was diagnosed when an animal’s body weight was two standard deviations less than the mean body weight of the control group. The methods of the preservation and treatment of the sample in mice were similar to the previous procedure.

### Immunohistochemistry and immunofluorescence measurement

Placental tissues embedded with paraffin were cut into 5-μm-thick slices along the longitudinal axis. Then, the slices were deparaffinized and rehydrated with xylene and a series of grades of alcohol and then were performed using a microwave treatment for 15 min in citrate buffer (pH 6.0). Then, they were soaked in 3% H_2_O_2_ for 25 min for immunochemistry or socked in Triton X-100 to penetrate the membrane for 30 min for immunofluorescence. The antigen retrieval sections were then blocked with 10% goat serum at 37°C for 30 min and incubated with a primary anti-P-gp (1:200) or anti-YB-1 (1:150) antibody overnight at 4°C, following by incubation with the secondary antibody at 37°C for 50 min. Slides were stained with DAB and counterstained with hematoxylin for immunochemistry or dyed with DAPI for immunofluorescence. The mean of integrated optic density (IOD) was measured in 6 different fields for each sample, 5 different samples of each group using Image-Pro Plus (version 6.1, Media Cybernetics, Silver Spring, MD, USA).

### Isolation of villous trophoblasts

A method of modified Kliman [34] and Zhang [35] was used for placental trophoblasts isolation and purification. Briefly, the tissue removed from the mother’s side of the placenta was minced after washing with normal saline and was digested with 0.125% trypsin and 0.03% DNase I four times (20, 20, 15, 10 min), followed by digestion termination with 10% fetal bovine serum. Purified trophoblast cells could be obtained by screening with nylon net (40 μm) after isolation using a 5–65% Percoll. Collected trophoblasts were seeded with a density at 2×10^6^ cells were inserted into 6-well plates.

### Cell culture and treatment

The BeWo cell line is derived from human placental villous carcinoma, which was purchased from the China Center for Type Culture Collection (Wuhan, China). The BeWo cells or placental trophoblasts were cultured in modified Eagle’s medium supplemented with 10% fetal bovine serum and 0.1% penicillin/streptomycin at 37°C in a 5% CO_2_ humidified incubator. The cells were treated with different concentrations of caffeine (0, 0.1, 1, 10, and 10 μM) for 48 h to test the effect of caffeine on P-gp expression.

To confirm the signaling pathway, nonspecific and competitive ryanodine receptor (RYR) antagonist (dantrolene), JNK inhibitor (SP600125), and YB-1 overexpression plasmid were used. Briefly, the BeWo cells were plated at a density of 4×10^5^ cells per well in 6-well plates, after reaching 30–50% confluent, dantrolene (10 μM), and SP600125 (10 μM) were added to the cells, respectively. As to the overexpression of YB-1, after being cultured in 6-well plates, BeWo cells were transfected with the plasmid 1 μg per well using Lipofectamine 3000 according to the manufacturer’s protocols. After 48 h transfection, the cells were harvested for the subsequent analysis.

### P-gp activity measurement

After incubation with caffeine for 48 h in a 96-well plate, the BeWo cells were washed by PBS (37°C) 3 times and then treated with 10 μM Rho 123 for 1.5 h. To detect the accumulation of Rho 123, cells were perforated with 1% Triton X-100 for 10 min after being washed with cold PBS. The fluorescence of Rho 123 accumulation was measured with a fluorescence plate reader (Molecular Devices, Wokingham, UK) (excitation/emission wavelength: 485 nm/530 nm).

### Transport experiment

The BeWo cells were transferred to transwell® polycarbonate membranes (12-mm diameter, 0.4-μm pore size). The cells were plated at a density of 1×10^5^ cells/cm^2^ with a certain volume of medium in the apical and basal chambers, and the medium was replaced daily. The cells were cultured with different concentrations of caffeine for 48 h. On day 6 of post-seeding, the confluent monolayer was formed according to the study’s findings [36]. 200 nM cortisol was added to the apical chamber, and the transwell was incubated at 37°C for 120 min. Then, a sample of 0.2 ml was taken from the basolateral compartment for cortisol measurement.

### Serum, placenta, and cell culture fluid corticosterone/cortisol measurement

Placental tissues were homogenized with 1ml PBS, then centrifuged at 12000×*g* at 4°C for 5 min. The levels of rats/mice serum and placental corticosterone were detected by an ELISA kit following the manufacturer’s introductions. The minimum detection of the corticosterone is 0.56 ng/mL. The intra-assay and inter-assay coefficients of variation were 4% and 5.5%. The cortisol levels of the human placenta and cell culture were tested using the ELISA kit.

### Total RNA extract and qRT-PCR

The total RNA was isolated using TRIzol reagent. Then, 1 μg of purified RNA was reverse-transcribed into cDNA using the qRT-PCR reverse kit according to the supplier’s instructions. The relative mRNA level of ABCB1 (ATP binding cassette subfamily B member 1), RYRs, and P300 was detected by an SYBR qPCR master mix kit. GAPDH was selected for rat and human and ubiquitin C (UBC) was selected for mouse as the housekeeping gene, and the relative expression levels of these genes were calculated using the 2^-ΔΔCt^ method. The sequences of primers for each gene are shown in Table 1. All oligonucleotide primers were synthesized by Sangon Biotech Co., Ltd. (Shanghai, China).

**Table 1:** Oligonucleotide primers in quantitative real-time PCR

Gene	Forward primer	Reverse primer	Locus
Rat			
abcb1a	TAGCAGGAGTGGTTGAAATG	CAAGCTCTGGGCATACATAG	NM_133401.2
abcb1b	CATCCAGAACGCAGACTTGA	CAGCCTGAACCATCGAGAAA	NM_012623.3
RYR1	CATCCTTTCATCCGTCACTC	TCATCTTCGCTCTTGTTGTAG	XM_039100851.1
RYR2	AGGGAGAGAGGAAGCCATTA	GGTCACTGAGACCAGCATTT	XM_039096071.1
RYR3	CACTGACAACTCCTTTCTCTAC	GGATCGTCCTCAAGGTCTTA	XM_039106806.1
GAPDH	GCAAGTTCAACGGCACAG	GCCAGTAGACTCCACGACA	NM_017008.4
Mouse			
abcb1a	CAGCCAGCATTCTCCGTAATA	GTGAGGATCTCTCCAGCTTTG	NM_011076.3
abcb1b	TCCCTGTTCTTTCTGGTTATGG	CCCGAGGTTTGCTACATTCT	NM_011075.2
UBC	AGGTGGGATGCAGATCTTTG	CCTCCTTGTCCTGGATCTTTG	NM_019639.4
Human			
ABCB1	GGTGGTGTCACAGGAAGAGATT	TCTAACAAGGGCACGAGCTATG	NM_001348945.2
RYR1	GCTCCCTGTGTGTGTGTAAT	CGGATGCTGGTGACATAGTT	NM_000540.3
RYR2	GAGATGGTCCCTCACCAAATAG	CGTCCCAAGAGGTCAATCAA	NM_001035.3
RYR3	GGAGAAGGTCAGCATAGACAAG	TCCAGTCACCACTTCAAACTC	NM_001036.6
YB-1	GCAGCAGACCGTAACCATTAT	TCTCCGATCCCTCGTTCTTT	NM_004559.5
P300	CCAGCCATGCAGAACATGAA	CGGAATTGTGAAGGCATGGT	NM_001429.4
GAPDH	GAAATCCCATCACCATCTTCCAG	ATGAGTCCTTCCACGATACCAAAG	NM_002046.7

### Chromatin immunoprecipitation (ChIP) and re-ChIP assays

To analyze binding levels of YB-1, P300, H3K9ac, and H3K14ac at the promoter region of the ABCB1 gene, we performed ChIP assays according to the manufacturer’s procedures. The samples were resuspended in 0.5 ml lysis buffer containing protease inhibitors and then sonicated. 10 μL lysis buffer of the samples was used for input DNA, and the left lysis buffer was incubated with Protein A/G beads used for immunoprecipitation with 1 μg anti-YB-1, anti-P300, anti-acetyl H3K9, anti-acetyl H3K14, and IgG antibody at 4°C overnight, followed by incubation with BSA-treated Proteinase K at 65°C for 8 h. For re-ChIP, an anti-YB-1 antibody was used for primary IP, followed by anti-P300 for the second IP, and a nonspecific IgG antibody was used as a control. The sheared DNA recovered from cross-linking was extracted with a DNA purification kit. The DNA associated with target proteins was analyzed by quantitative PCR to quantify the amount of ABCB1 DNA associated with these marks. Data were obtained by normalizing 2^-ΔΔCt^ from qRT-PCR. ChIP primers spanning the ABCB1 binding region used are as follows: Human (ABCB1): TGTAGCTGGTTGGTTGGGAT and CTGGCCTTGTGACTTGCTTT (histone acetylation); TCTCGAGGAATCAGCATTCA and AAGAGCCGCTACTCGAATGA (YB-1 and P300); Rat (abcb1a): TTATGAAGTGTGCGGGAGTG and GGACCGTAGCGAGAACAAAT (histone acetylation); Rat (abcb1b): GGAGCGCCATGTAAAATGCA and CGTAGCGAGAACAAATGCCA (histone acetylation).

### Protein extract, Western blotting, and co-immunoprecipitation assays

Homogenate tissues or cells were lysed in RIPA buffer containing 1% Protease Inhibitor Cocktail. For the nuclear and cytoplasm protein fractionation, a nuclear and cytoplasmic protein preparation kit was used following the manufacturer’s protocol. The protein concentration of the samples was determined with a BCA assay kit. Equal amounts of protein were separated by SDS-polyacrylamide gel electrophoresis (SDS-PAGE) and transferred onto a PVDF microporous membrane. The membranes were blocked with 5% skim milk and then incubated with anti-P-gp (1:1000), anti-YB-1 (1:1000), anti-P300 (1:1000), anti-JNK (1:1500), anti-p-JNK (1:1000), anti-H3 (1:5000), or anti-GAPDH (1:2000) antibody at 4°C overnight, followed by incubation with secondary antibodies at room temperature for 1.5 h. The blots were visualized by the ECL kit. For the immunoprecipitation assay, the protein was incubated with 1 μg p-JNK or YB-1 antibody overnight. Then, 40 μl 50% protein A/G agarose beads were added and continued for 4 h. Beads were resuspended and boiled with 2× SDS-PAGE loading buffer for western blotting analysis.

### Statistical analysis

Data were presented as mean ± S.E.M. Statistical differences among gene expression in the placentas were calculated and plotted using Graph Pad Prism 6.0 (GraphPad Software, Inc., USA). Comparisons between 2 groups were made by two-tailed Student *t* tests and >2 groups were analyzed by one-way ANOVA method. The association of different targets was analyzed using Pearson methods. For all tests, a *P*<0.05 was considered to be significantly different.

## Results

### Correlations among P-gp expression in the human placenta, fetal cord blood cortisol levels, and neonatal birth weights

**Fig. 1:**
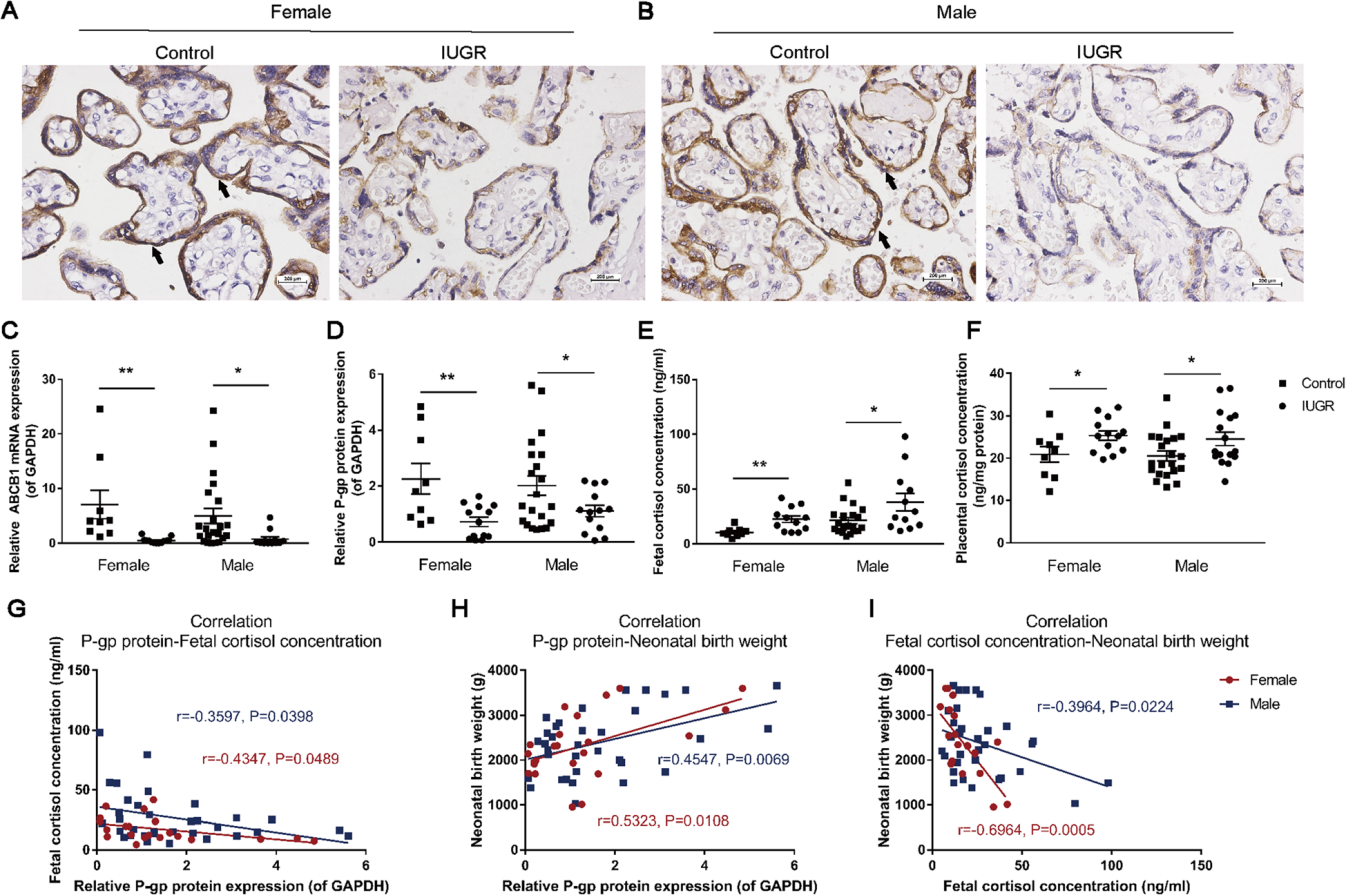
Placental P-glycoprotein (P-gp) expression and fetal cord serum cortisol concentrations in control and intrauterine growth retardation (IUGR) neonates. **A**, **B** Immunostaining of P-gp in the human female and male placenta (400×). **C**, **D** Relative mRNA and protein expression levels of P-gp in the female and male placentas; **E**, **F** Cortisol levels in the female (*n*=9 for control, *n*=13 for IUGR) and male (*n*=21 for control, *n*=13 for IUGR) placentas and fetal cord serum; **G** Correlations between placental P-gp protein levels and fetal cortisol concentrations; **H** Correlations between neonatal birth weights and placental P-gp protein levels; **I** Correlations between neonatal birth weights and fetal cord serum cortisol concentrations in females (*n*=22) and males (*n*=34). Mean ± S.E.M. ^*^P<0.05, ^**^P<0.01 vs*.* control

To figure out the relationship between placental P-gp expression and fetal birth weight, we collected placental and fetal cord blood samples of clinical IUGR and the gestational age-matched control neonates. The clinical characteristics of the included patients are shown in Table 2. Compared with the control group, there was no noticeable difference in the age and gestational age of pregnant women in the IUGR group, while the body length and body weight of the neonates were significantly reduced (*P*<0.01). Using immunohistochemical analysis, we found that P-gp was mainly expressed on maternal-facing syncytiotrophoblasts (black arrow, Fig. 1A, B). It was further found that the mRNA and protein expression levels of placental P-gp (ABCB1) were significantly reduced, and the cortisol concentrations of the placenta and fetal cord blood were increased in the IUGR group, and there was no significant difference between males and females (*P*<0.05, *P*<0.01, Fig. 1C–F). Additionally, the placental P-gp protein expression levels in female and male neonates were negatively correlated with fetal cord blood cortisol levels and positively correlated with birth weights, while the fetal cord blood cortisol levels were negatively correlated with the birth weights in male and female neonates (*P*<0.05, *P*<0.01, Fig. 1G–I). These results suggest that placental P-gp expression was decreased, the fetal cord blood cortisol level was increased in the neonates with IUGR, and the birth weight was significantly related to placental P-gp expression and cord blood cortisol level.

**Table 2:** Clinical characteristics of neonates

Group	Female		Male	
	Control (*n*=9)	IUGR (*n*=13)	Control (*n*=21)	IUGR (*n*=13)
Maternal age	34.0 ± 2.7	30.0 ± 1.3	32.0 ± 1.5	32.0 ± 1.3
Gestational weeks	36.0 ± 0.8	35.0 ± 0.7	36.0 ± 0.4	35.0 ± 0.7
Neonatal birth weight (g)	3041 ± 159	1863 ± 128 ^**^	2769 ± 130	1793 ± 115 ^**^
Neonatal birth length (cm)	50.0 ± 0.4	42.0 ± 1.6 ^**^	48.0 ± 0.6	43.0 ± 1.1 ^**^

*IUGR* intrauterine growth retardation, ^**^P<0.01 vs*.* control

### Changes of fetal body/placental weights, fetal serum/placental corticosterone levels, and P-gp expression and their relationships in PCE-induced IUGR rat model

**Fig. 2:**
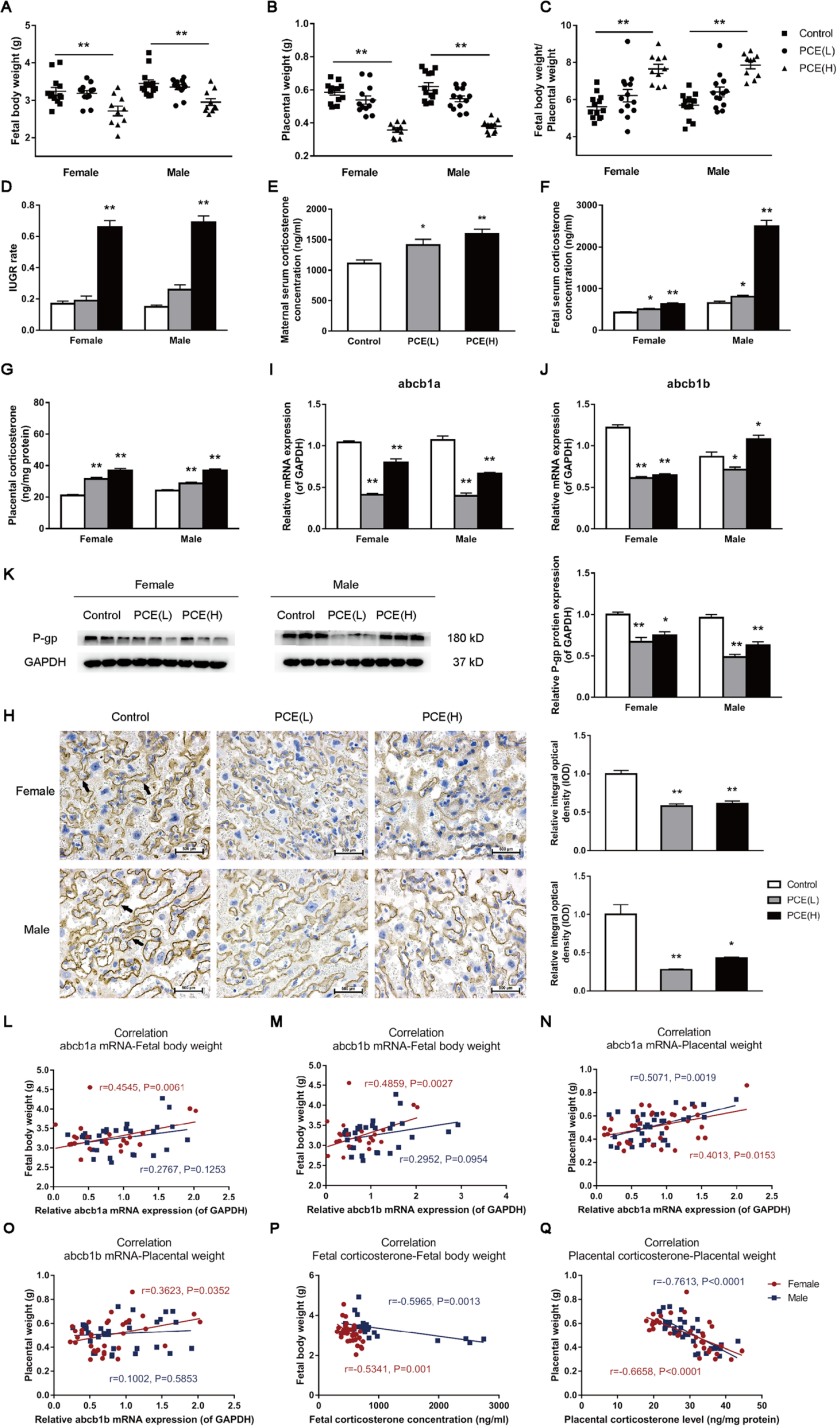
Changes of fetal body/placental weights, fetal serum/placental corticosterone levels, and placental P-glycoprotein (P-gp) expression in prenatal caffeine exposure (PCE)-related intrauterine growth retardation (IUGR) rat model. **A** Fetal body weight; **B** placental weight; **C** fetal body weight/placental weight; **D** IUGR rate; **E**–**G** corticosterone levels in maternal serum, fetal serum, and placentas; **H** immunostaining of P-gp (400×), *n*=5; **I**, **J** relative mRNA expression levels of ATP-binding cassette, sub-family B, member 1a (abcb1a) and abcb1b, *n*=10–12; **K** protein level of placental P-gp, *n*=3; **L**, **M** correlations between abcb1a or abcb1b mRNA expression levels and fetal body weights; **N**, **O** correlations between abcb1a or abcb1b mRNA expression levels and placental weights; **P** correlations between fetal body weights and fetal serum corticosterone concentrations; **Q** correlation between placental weights and placental corticosterone levels. *n*=34. Mean ± S.E.M. ^*^P<0.05, ^**^P<0.01 vs. control

To verify the relationship between P-gp and the occurrence of IUGR in population experiments, pregnant Wistar rats were intragastrically given 30 and 120 mg/kg·d caffeine during GD 9-20 to establish an in vivo IUGR model. As shown in Fig. 2, compared with the control group, the fetal body weights and placental weights of the PCE(L) and PCE(H) group were decreased, and the ratio of fetal body weights/placental weights and IUGR rate were increased (*P*<0.01, Fig. 2A–D); the corticosterone levels in maternal serum, fetal serum, and placenta were significantly increased (*P*<0.05, *P*<0.01, Fig. 2E–G). P-gp is encoded by a single gene (ABCB1) in humans, while there are two genes (abcb1a and abcb1b) that are required to encode this protein in rodents [37]. Compared with the control group, the mRNA and protein levels of P-gp were significantly downregulated in the PCE group according to the qRT-PCR, Western blotting, and immunohistochemical analysis, and there was no significant difference in males and females (*P*<0.05, *P*<0.01, Fig. 2H–K). These results indicated the reduced fetal body weights, increased corticosterone levels in the placenta and fetal serum, and the inhibited placental P-gp expression in the PCE-induced IUGR rat model.

Next, we found that both abcb1a and abcb1b mRNA expression levels were positively correlated with fetal body weights in females (*P*<0.05, *P*<0.01, Fig. 2L) but not in males (Fig. 2M). We also found a positive correlation between abcb1a expression levels and placental weights in females and males (*P*<0.05, *P*<0.01, Fig. 2N). Additionally, abcb1b expression levels were positively correlated with placental weights in females (*P*<0.05) but not in males (Fig. 2O). Meanwhile, we observed that fetal serum corticosterone levels were negatively correlated with fetal body weights and the placental corticosterone levels were negatively correlated with placental weights (*P*<0.05, *P*<0.01, Fig. 2P, Q). These results indicate that the inhibition of fetal body/placental weights was not only associated with the reduction of P-gp expression but also the upregulation of fetal serum/placental glucocorticoid levels.

### Changes of placental RYR/JNK/YB-1 pathway and histone acetylation of the abcb1a/b promoter in PCE-induced IUGR rat model

**Fig. 3:**
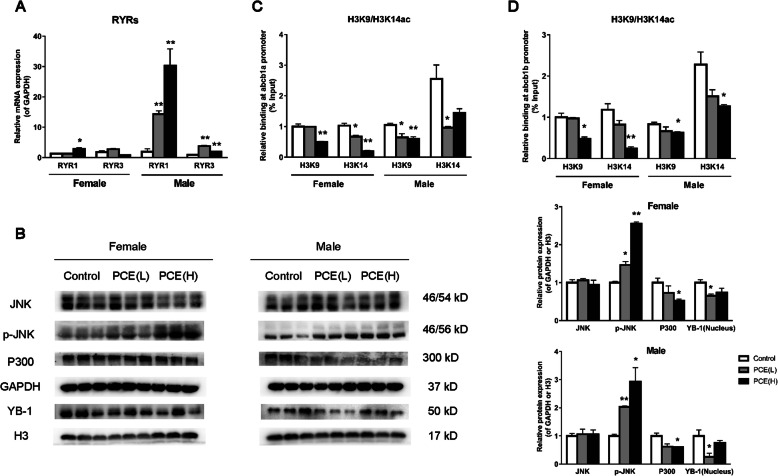
Changes of placental ryanodine receptor/C-Jun N-terminal kinase/Y-box protein 1 (RYR/JNK/YB-1) pathway and histone acetylation of ATP-binding cassette, sub-family B, member 1a/b (abcb1a/b) promoter region of prenatal caffeine exposure-induced intrauterine growth retardation rat model. **A** Relative mRNA expression levels of RYRs, *n*=10–12; **B** Relative protein expression of placental JNK, p-JNK, and E1A binding protein P300 (P300) was standardized by GAPDH, and relative protein expression of nucleus YB-1 was standardized by H3, *n*=3; **C**, **D** Histone 3 Lysine 9 (H3K9) and histone 3 Lysine 14 (H3K14) acetylation levels in the promoter region of abcb1a and abcb1b, *n*=3. Mean ± S.E.M. ^*^P<0.05, ^**^P<0.01 vs. control

To verify the molecular mechanism of the inhibitory effect of PCE on P-gp expression, we detected the expression levels of 3 subtypes of RYR and the phosphorylation of JNK. The results showed that RYR1 and RYR3 mRNA expression levels were increased in the PCE group, as compared with the control group (*P*<0.05, *P*<0.01, Fig. 3A), while RYR2 was not detected in the rat placenta. Besides, there was no significant change in total protein expression but increased phosphorylation of JNK in the PCE group (*P*<0.05, *P*<0.01, Fig. 3B). Then, we further carried out the relevant experiments and found that the protein level of YB-1 in the nucleus of the PCE group was decreased (*P*<0.05, Fig. 3B). These findings indicated that the placental RYR1/3 expression levels were increased, the JNK signal was activated and the YB-1 nuclear translocation was inhibited in the PCE-induced IUGR rat model, and there was no significant difference in males and females.

To determine whether the epigenetic modifications of ABCB1 are involved in the inhibitory effect of PCE on P-gp expression, we further performed ChIP experiments. The results showed that H3K9ac and H3K14ac levels of placental ABCB1 were significantly reduced in PCE-related IUGR rats, as compared with the control group (*P*<0.05, *P*<0.01, Fig. 3C, D). P300 is a kind of acetyltransferase that plays an essential role in regulating the acetylation modifications in the gene promoter. Compared with the control group, the protein level of placental P300 was significantly decreased in the PCE-related IUGR group (*P*<0.05, Fig. 3B). These results indicated that the placental p300 expression and the histone acetylation levels of the abcb1a and abcb1b promoter were reduced in the PCE-induced IUGR rat model, and there was no significant difference between the two sexes.

### Changes of P-gp expression, cortisol efflux, and related pathway in caffeine-treated placental trophoblasts

**Fig. 4:**
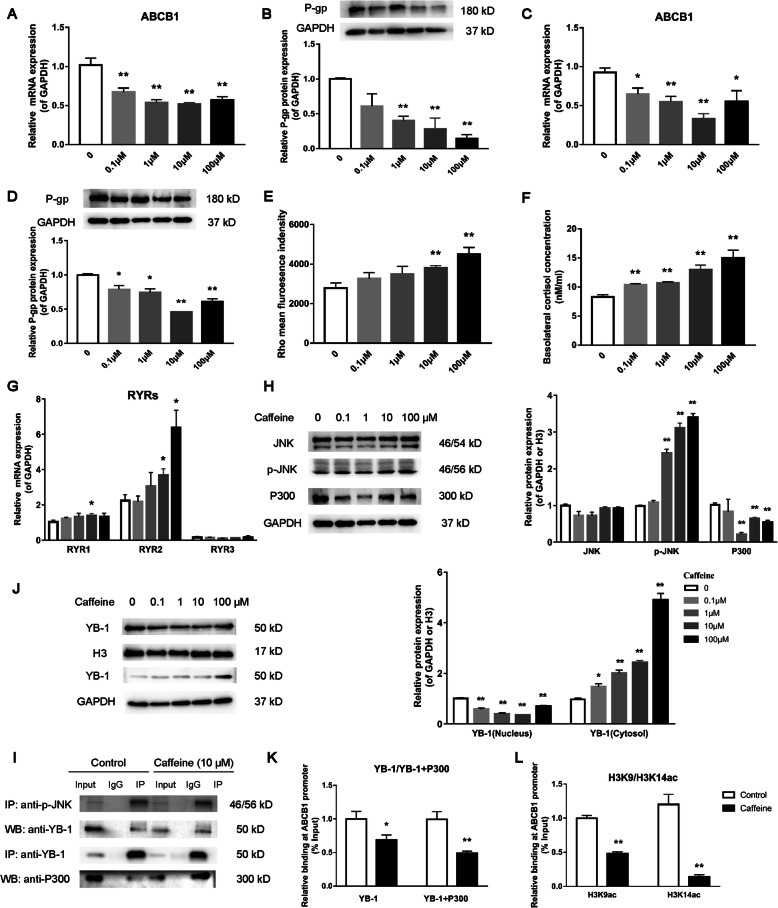
Effects of caffeine (0–100 μM) on P-glycoprotein (P-gp) expression, cortisol transport, and related pathway in placental trophoblasts. **A**, **B** Relative mRNA (*n*=6) and protein (*n*=3) expression levels of P-gp in primary placental trophoblasts; **C**, **D** relative mRNA (*n*=6) and protein (*n*=3) expression levels of P-gp in BeWo cells; **E** Rho 123 accumulation was analyzed by the fluorescence plate reader, *n*=6; **F** Effect on cortisol efflux in BeWo cells by caffeine treatment, *n*=6; **G** relative mRNA expression levels of RYRs, *n*=6; **H** protein levels of JNK, p-JNK, and E1A-binding protein P300 (P300), *n*=3; **I** Protein-protein interactions between p-JNK and YB-1 and between YB-1 and P300; **J** Relative protein levels of the nucleus and cytosol YB-1, *n*=3; **K** binding level of YB-1 on the ABCB1 promoter region; binding level of YB-1 and P300 on the ABCB1 promoter region; **L** histone 3 Lysine 9 (H3K9) and histone 3 Lysine 14 (H3K14) acetylation levels on the ABCB1 promoter region, *n*=3. Mean ± S.E.M. ^*^P<0.05, ^**^P<0.01 vs. control

To confirm the inhibitory effect of caffeine on P-gp expression in placental trophoblast cells, the primary trophoblasts extracted from the human placenta and the human trophoblast cell line BeWo cells were used for in vitro experiments. After treatment with different concentrations of caffeine (0.1, 1, 10, 100 μM) for 48 h, it was found that the mRNA and protein expression levels of P-gp were significantly reduced in the above two types of cells (*P*<0.05, *P*<0.01, Fig. 4A–D). Rho 123 belongs to the substrate of P-gp, and its fluorescence accumulation can reflect the transport activity of P-gp [38]. We found an increased rho 123 accumulation by caffeine in a concentration-dependent manner in BeWo cells (*P*<0.01, Fig. 4E), indicating that caffeine reduced P-gp activity. To prove the effect on the cortisol efflux transport capacity of trophoblasts by caffeine, we used an in vitro model of the placental barrier constructed by BeWo cells using the Transwell chamber [39]. After the adding of cortisol in the upper chamber, the cortisol concentration was increased in the lower chamber after caffeine treatment (*P*<0.01, Fig. 4F), indicating the inhibited cortisol efflux capacity of P-gp by caffeine.

To further elucidate the molecular mechanism of P-gp expression inhibition, we tested the relevant indicators in the BeWo cells after caffeine treatment. Compared with the control group, the mRNA expression levels of RYR1 and RYR2 were increased in the caffeine group (*P*<0.05, Fig. 4G), while RYR3 expression level did not change significantly. And no apparent change in the protein expression of JNK but noted increased p-JNK protein level after caffeine treatment (*P*<0.01, Fig. 4H). Co-IP results showed that p-JNK can interact with YB-1 (Fig. 4I). We found that compared with the control group, the protein level of nuclear YB-1 was decreased (*P*<0.05, *P*<0.01, Fig. 4J) and the binding level of YB-1 on the ABCB1 promoter was decreased in the caffeine group (*P*<0.01, Fig. 4K). These findings indicated that caffeine can induce the RYR1/2 and p-JNK expression, increase the interaction between p-JNK and YB-1, and reduce the YB-1 nuclear translocation, thereby inhibiting the binding of YB-1 on the ABCB1 promoter.

Furthermore, the protein level of P300 was decreased (*P*<0.01, Fig. 4H). We also observed an interaction between YB-1 and P300 in the nucleus of BeWo cells both in the control and caffeine-treated group by Co-IP experiments (Fig. 4I). Using Re-ChIP experiments, we further found that YB-1 and P300 bound to the same region of ABCB1 promoter, and the binding level of YB-1-P300 complex was downregulated after caffeine treatment (*P*<0.01, Fig. 4K). Besides, the H3K9 and H3K14 acetylation levels on the ABCB1 promoter were reduced in BeWo cells treating with caffeine (*P*<0.01, Fig. 4L). These results indicated the reduced recruitment of P300 by YB-1 by caffeine, which could further reduce the H3K9ac and H3K14ac levels on the ABCB1 promoter and inhibit P-gp expression.

### Effects of RYRs, JNK inhibition, and YB-1 overexpression on P-gp expression and cortisol efflux function in BeWo cells

**Fig. 5:**
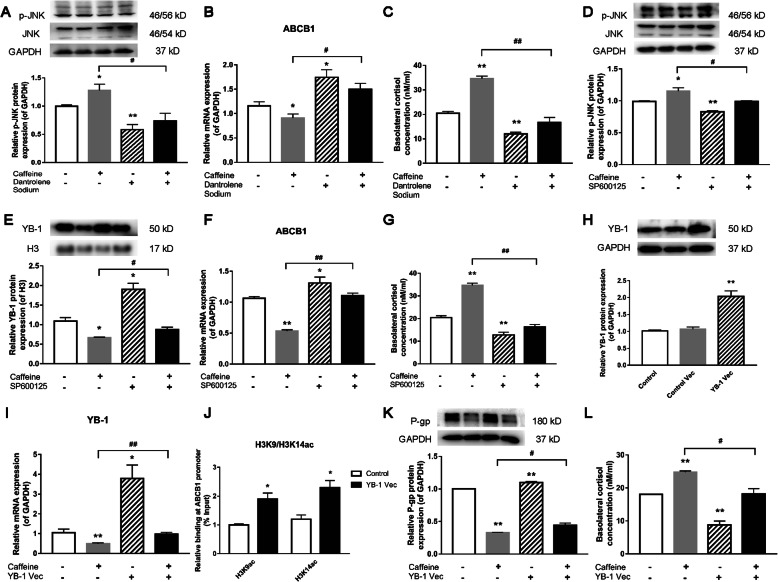
Effects of dantrolene, SP600125, and Y-box protein 1 (YB-1) overexpression on P-glycoprotein (P-gp) expression and cortisol efflux function in BeWo cells. **A**, **B** Relative mRNA expression levels of ATP-binding cassette, sub-family B, member 1 (ABCB1), and protein levels of C-Jun N-terminal kinase (JNK)/p-JNK treating with 10 μM caffeine and/or 10 μM dantrolene; **C** effect of caffeine and/or dantrolene on cortisol efflux, *n*=6; **D**–**F** relative protein levels of JNK/p-JNK, nucleus YB-1, and relative mRNA expression level of ABCB1 after treatment with 10 μM caffeine and/or 10 μM SP600125; **G** effect of caffeine and/or SP600125 on cortisol efflux, *n*=6; **H** relative YB-1 protein level after transiently transfected with YB-1 plasmid or control empty plasmid; **I** relative mRNA expression of YB-1 after treatment with 10 μM caffeine and/or YB-1 transfection, *n*=6; **J** histone 3 Lysine 9 (H3K9) and histone 3 Lysine 9 (H3K14) acetylation changes on ABCB1 promoter after YB-1 overexpression, *n*=3; **K**, **L** effect of caffeine and/or YB-1 overexpression on P-gp protein expression (*n*=3) and cortisol efflux (*n*=6). Mean ± S.E.M. ^*^P<0.05, ^**^P<0.01 vs. control; ^*#*^*P*<0.05, ^*##*^*P*<0.01 vs. 10 μM caffeine

To further verify that RYRs, JNK, and YB-1 are involved in the P-gp regulation, we observed the effects on P-gp expression and related pathways by caffeine (10 μM) alone or combined with dantrolene sodium (RYR antagonist, 10 μM), SP600125 (JNK inhibitor, 10 μM), and YB-1 plasmid transfection. As shown in Fig. 5, dantrolene sodium significantly reversed the caffeine-induced JNK phosphorylation, P-gp expression inhibition, and cortisol efflux (*P*<0.05, *P*<0.01, Fig. 5A–C). SP600125 significantly inhibited the JNK phosphorylation and reversed the inhibitory effect of caffeine on the protein level of nuclear YB-1 and P-gp and the efflux of cortisol (*P*<0.05, *P*<0.01, Fig. 5D–G). Next, the mRNA and protein expression levels of YB-1 were increased after the transfection of the YB-1 overexpression plasmid (*P*<0.01, Fig. 5H, I). The overexpression of YB-1 increased the H3K9ac and H3K14ac levels on the ABCB1 promoter (*P*<0.05, Fig. 5J). The P-gp expression inhibition and the cortisol efflux by caffeine can be reversed by the overexpression of YB-1 (*P*<0.05, *P*<0.01, Fig. 5K, L). These results indicated that the RYR/JNK/YB-1 pathway is involved in the P-gp expression suppression and cortisol efflux function by caffeine through epigenetic regulation.

### Effect of P-gp inducer sodium ferulate on fetal weight loss caused by PCE

To confirm the regulation of P-gp on fetal weight loss, we observed the potential therapeutic effects of P-gp inducer sodium ferulate on fetal and placental weights induced by PCE. Both caffeine (120 mg/kg·d) and sodium ferulate (50 mg/kg·d) were administered from GD9 to GD18 by gavage. As shown in Table 3, compared with the control, maternal weight gain rate, the male and female fetal and placental weights were significantly reduced in the caffeine groups (*P*<0.05, *P*<0.01), but not significantly changed in the sodium ferulate groups. However, sodium ferulate could reverse the inhibitory effects of caffeine on the maternal body weight gain rate (*P*<0.05) as well as the male and female fetal body and placental weights (*P*<0.05, *P*<0.01). These results suggest that sodium ferulate can reverse the inhibitory effects of caffeine on the fetal body and placental weights.

**Table 3:** Effect of P-gp inducer sodium ferulate on maternal weight gain rate, fetal body, and placental weights

Group	Maternal weight gain rate (%)	Fetal body weight (g)		Placental weight (g)	
		Female	Male	Female	Male
Control	52.0±1.5	1.100±0.024	1.100±0.026	0.085 ± 0.0037	0.086 ± 0.0031
Caffeine	40.0±1.7^**^	0.850±0.026 ^*^	0.870±0.030 ^*^	0.072 ± 0.003 ^**^	0.074 ± 0.0025 ^**^
Sodium ferulate	52.0±1.5	1.100±0.063	1.200±0.062	0.090±0.0031	0.095 ± 0.0039
Caffeine + sodium ferulate	47.0±2.8^**#**^	0.990 ±0.062 ^**##**^	1.000±0.053 ^**##**^	0.088±0.0029 ^**#**^	0.093 ± 0.0028 ^**#**^

^*^P<0.05, ^**^P<0.01 vs. control; ^*#*^*P*<0.05, ^*##*^*P*<0.01 vs. caffeine

**Fig. 6:**
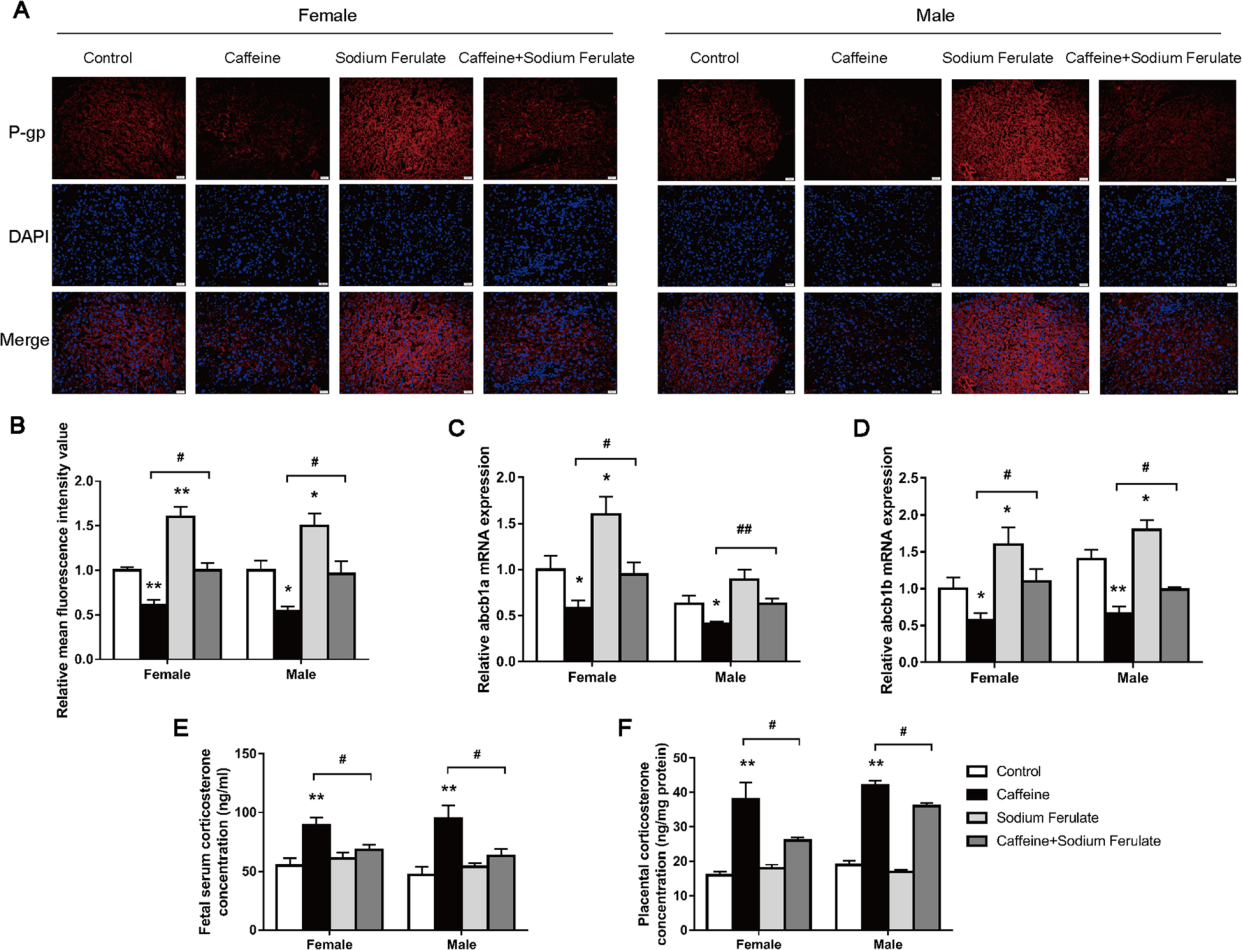
Effect of sodium ferulate on placental P-glycoprotein (P-gp) expression, placental, and fetal serum corticosterone levels. **A** Representative photomicrographs of immunofluorescence for P-gp in female and male placenta (200×); **B** relative mean fluorescence intensity value of P-gp, *n*=3; **C**, **D** relative mRNA expression levels of ATP-binding cassette, sub-family B, member 1a (abcb1a) and abcb1b, *n*=8–10; **E**, **F** Placental and fetal serum corticosterone levels, *n*=8–10. Mean ± S.E.M. ^*^P<0.05, ^**^P<0.01 vs*.* control; ^*#*^*P*<0.05, ^*##*^*P*<0.05 vs. caffeine

Further, as shown in Fig. 6A–D, the protein expression levels of P-gp, as well as the mRNA of abcb1a/b, were reduced in the caffeine groups (*P*<0.05, *P*<0.01) but increased in the sodium ferulate groups in both the male and female placentas, as compared with the control groups. Moreover, sodium ferulate could reverse the inhibitory effects of caffeine on P-gp expression in both male and female placentas (*P*<0.05, *P*<0.01, Fig. 6A–D). As for the placental and fetal serum corticosterone levels, they were increased in the caffeine groups (*P*<0.05, *P*<0.01) but were not significantly changed in the sodium ferulate groups, as compared with the control groups (Fig. 6E, F). Sodium ferulate could also reverse the enhancing effects of caffeine on the male and female placental and fetal serum corticosterone levels (*P*<0.05, *P*<0.01, Fig. 6E, F). These results suggest that sodium ferulate can reverse the inhibitory effects of caffeine on the placental P-gp expression and upregulation of the fetal serum/placental glucocorticoid levels.

### Correlations between JNK/YB-1/P300 pathway and P-gp expression in the human placentas

**Fig. 7:**
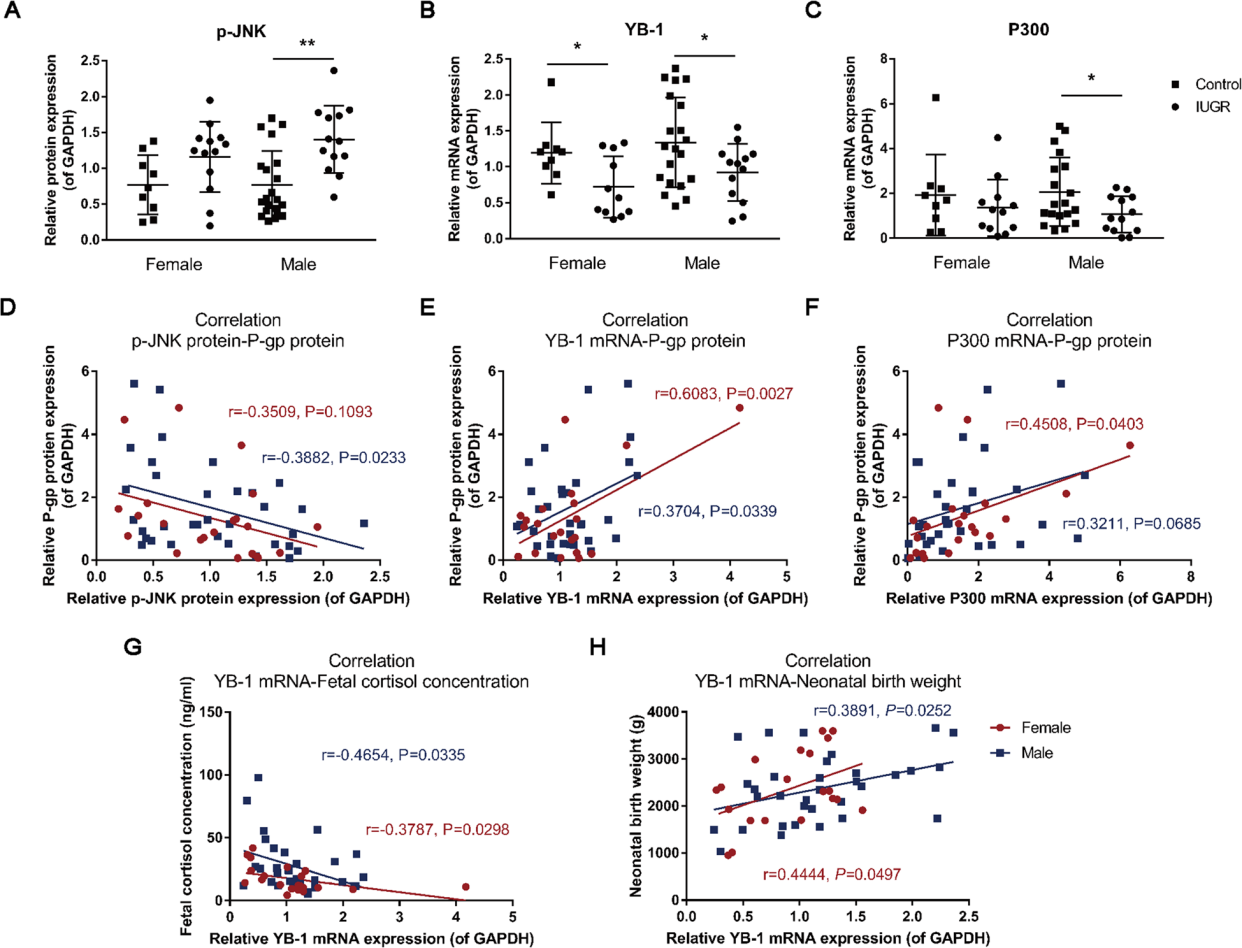
Expression of placental C-Jun N-terminal kinase (JNK)/Y-box protein 1(YB-1) pathway in intrauterine growth retardation (IUGR) and the gestational age-matched control neonates. Relative mRNA or protein expression levels of p-JNK (**A**), YB-1 (**B**), and P300 (**C**) in the female and male placentas, *n*=9–21; **D**–**F** correlations between placental P-gp protein levels and p-JNK, YB-1, and P300 expression levels in the female and male placenta; **G**, **I** correlations between fetal cord cortisol concentrations, neonatal birth weights, and YB-1 mRNA expression levels in female and male placenta, *n*=22–34. Mean ± S.E.M. ^*^P<0.05, ^**^P<0.01 vs*.* control

Finally, we examined the changes of the JNK/YB-1/P300 pathway in the human placentas to confirm their involvement in placental P-gp regulation of IUGR neonates. Compared with the control, the protein level of placental p-JNK in the male neonates was significantly increased, while the mRNA expression level of YB-1 in both female and male neonates, as well as P300 in the male neonates, were significantly decreased in the IUGR group (*P*<0.05, *P*<0.01, Fig. 7A–C). Furthermore, we observed that the placental p-JNK protein levels in the male neonates were negatively correlated with P-gp protein levels, while the mRNA expression levels of placental YB-1 in both female and male and P300 in female neonates were positively correlated with P-gp protein levels (*P*<0.05, *P*<0.01, Fig. 7D–F). Moreover, the placental YB-1 expression levels were negatively correlated with fetal cord blood cortisol concentrations, while positively correlated with neonatal birth weights in female and male neonates (*P*<0.05, *P*<0.01, Fig. 7G–I). These results suggest that the placental JNK/YB-1/P300 pathway may participate in the occurrence of IUGR mediated by P-gp expression inhibition.

## Discussion

### The occurrence of IUGR was associated with P-gp-mediated placental glucocorticoid barrier opening

A large number of previous studies in our lab have demonstrated that adverse environment during pregnancy (such as xenobiotics exposure and food intake restriction) can increase maternal and fetal glucocorticoid levels and partially compensate the fetal function development (thrifty phenotypic programming) to help the fetus get through the dangerous intrauterine period [40, 41]. However, such excessive maternal glucocorticoid exposure of the fetus can cause a higher risk of IUGR occurrence and an enhanced offspring’s susceptibility to multiple adult diseases [41–43]. Clinical studies have shown that the fetal cord blood corticosteroid levels were negatively correlated with fetal body weights [15]. It is known that the placental glucocorticoid barrier plays an important role in protecting the fetus from excessive exposure to maternal glucocorticoids. As one of the “placental glucocorticoid barriers,” P-gp is mainly expressed in the apical membrane of syncytiotrophoblasts, which can be in contact with maternal blood directly [44, 45]. Studies have shown that the P-gp can efflux exogenous and endogenous substrates and mediate signal transduction and is involved in maintaining the normal structure and function of the placenta [46–48]. However, the relationship between placental P-gp and IUGR is still unclear. In this study, P-gp expression in the human placenta was significantly reduced, and the placental and fetal cord blood cortisol levels were increased in the IUGR group. Besides, the neonatal birth weight was positively correlated with the placental P-gp protein levels and negatively correlated with fetal cord blood cortisol levels. The results of the above human studies suggest that fetal weight loss is likely to be related to placental P-gp expression suppression and fetal overexposure to maternal glucocorticoids.

Epidemiological surveys pointed out that a lot of pregnant women consume caffeine worldwide, and the caffeine intake of some pregnant even exceeded 300 mg/d [49–51]. Evidence from WHO epidemiological studies demonstrated that caffeine consumption exceeding 300 mg/d will increase the risk of IUGR occurrence, and the developmental toxicity increased with caffeine intak e[52, 53]. Previous studies in our laboratory have found that daily dose of caffeine exposure [20 mg/kg·d in rats, equivalent to 194 mg of caffeine per day for a 60 kg woman according to the dose conversion between humans and rats (1:6.17 )] can cause an increased rate of IUGR occurrence, dysplasia of multiple organs (including the hippocampus, adrenal gland, liver, bone), and susceptibility to multiple chronic diseases, such as non-alcoholic fatty liver, metabolic syndrome, and osteoarthritis, which are related to PCE-induced maternal glucocorticoid overexposure [16, 23–26]. In this study, by using the PCE-induced IUGR rat model, we analyzed the role of placental P-gp in mediating the IUGR occurrence and clarified its underlying mechanism. The results showed that the fetal body weight was significantly decreased and the IUGR rate was increased (female: 66% vs. 17%; male: 69% vs. 15%) in the PCE(H) group, indicating that the IUGR rat model was successfully established. Further, we found that PCE significantly reduced the mRNA and protein expression levels of placental P-gp and increased the corticosterone levels in the placental tissue and fetal serum. The correlation analysis indicated that the mRNA expression levels of placental abcb1a/b were positively correlated with placental/fetal weights, while the corticosterone levels in the placenta and fetal serum were negatively correlated with placental and fetal weights, respectively. These results were consistent with changes in multiple indicators of the clinical IUGR specimens.

BeWo cell is an *in vitro* model commonly used for studying placental function. Using the transport model constructed by BeWo cells, studies have found that overexpression of P-gp can restrict the passage of cortisol and dexamethasone [54, 55]. In this study, using the same transport model, we confirmed that caffeine can inhibit the P-gp expression and cortisol efflux of trophoblasts. All the above results indicate that PCE (caffeine) on the one hand increases maternal glucocorticoid level, and on the other hand, it opens the placental glucocorticoid barrier by inhibiting placental P-gp expression, thereby increasing the fetal glucocorticoid concentration and inhibiting fetal development. Previous studies have confirmed the impaired structure and function of the rat placenta in the PCE model [56]. It has also been reported that excessive glucocorticoids can impair placental development and function [12, 17]. The present study confirmed that PCE increased the glucocorticoid level in the placenta, and the excessive placental glucocorticoids can inhibit placental development and further affected fetal development. Noteworthy, although PCE reduced both the fetal body and placental weight, the ratio of fetal body/placental weight was increased. According to the characteristic change of “smaller placenta and bigger fetus,” we speculate that the fetal growth and development under PCE have been compensated to a certain extent, which is related to fetal multi-organ “thrifty phenotype” development caused by intrauterine glucocorticoid overexposure [42, 57].

### RYR/JNK/YB-1 pathway mediates the inhibition of P-gp expression in the placenta of IUGR rats induced by caffeine

Studies have shown that there are 3 subtypes of RYRs, and caffeine can increase the sensitivity of RYRs [58]. However, whether RYRs are involved in the regulation of placental P-gp by caffeine has not been reported. In the present study, we found that the rat placenta mainly expressed RYR1 and RYR3, while human trophoblasts mainly expressed RYR1 and RYR2. Caffeine increased the expression levels of RYR1 and RYR2 in the trophoblasts, while RYR antagonist can reverse the inhibitory effect of caffeine on P-gp expression and cortisol efflux. The studies have shown that the increased sensitivity of RYRs can reduce the threshold of spontaneous Ca^2+^ release from the endoplasmic reticulum [59]; the ozone in the spinal cord neurons can activate JNK/MAPK signaling by inducing the release of endoplasmic reticulum Ca^2+^, leading to neurotoxicity [60]. In this study, caffeine activated the JNK pathway, while inhibiting JNK can reverse the effect of caffeine on P-gp expression and cortisol efflux, indicating that the JNK pathway is involved in caffeine-induced inhibition of P-gp expression and opening of glucocorticoid barrier. YB-1 is an important member of the Y-box protein family, which can regulate gene transcription, translation, stress, and DNA repair [28]. Studies have shown that the overexpression of JNK1 or the activation of JNK signaling can inhibit YB-1 from entering the nucleus in MCF-7/Dox cells [27]. It is found that p-JNK can interact with YB-1 in BeWo cells in our study, while the inhibition of JNK can reverse the effect of caffeine on YB-1 nuclear translocation, indicating that p-JNK can bind to YB-1 and inhibit YB-1 from entering the nucleus after caffeine activates JNK signaling. We further found that caffeine can inhibit the binding of YB-1 to the ABCB1 promoter region. The inhibitory effect of caffeine on P-gp expression and cortisol efflux was canceled after YB-1 overexpression, suggesting that the reduction of YB-1 nuclear translocation mediates the transcriptional inhibition of P-gp and the opening of the placental glucocorticoid barrier induced by caffeine.

Recently, research on the epigenetic regulation of P-gp has been limited to studies about tumor [61]. However, its histone acetylation mechanism in the placenta has not been reported yet. A previous study demonstrated that drug-induced acetylation of apurinic/apyrimidinic endonuclease 1 can enhance the formation of the YB-1/p300 complex on the gene promoter in HEK-293T cells [62]. As an acetyltransferase, P300 plays an important role in regulating the epigenetic modification of genes. In this study, YB-1 can interact with P300, and the two can bind to the same ABCB1 promoter region, while caffeine can inhibit the binding of the YB-1-P300 complex to the ABCB1 promoter. Our study also shown that P300 expression, and ABCB1 (or abcb1a/b) promoter H3K9ac and H3K14ac levels were significantly reduced in the placenta in PCE-induced IUGR rats, as well as in BeWo cells treated with caffeine, and further overexpression of YB-1 can increase the ABCB1 promoter H3K9ac and H3K14ac levels. These results indicate that caffeine-induced decrease of YB-1 nuclear translocation can reduce the recruitment of P300 and inhibit the binding of the two proteins on the ABCB1 promoter, resulting in decreases of H3K9ac and H3K14ac levels of P-gp promoter and P-gp expression.

Taken together, PCE can inhibit the P-gp expression in placental trophoblast cells, and its underlying mechanism is related to the fact that caffeine can activate the RYR/JNK signaling, and promote the binding of p-JNK to YB-1, thereby inhibiting the expression of YB-1 and its nuclear translocation. The reduction of YB-1 in the nucleus, on the one hand, directly inhibits P-gp transcription; on the other hand, it inhibits the recruitment of P300 and reduces histone acetylation (H3K9 and H3K14) of the P-gp promoter, thus enhancing the inhibitory effect on the placental P-gp expression (Additional file 1: Figure S1).

### Potential early warning, prevention, and treatment target for IUGR

P-gp can prevent excessive exogenous and endogenous harmful substances from entering the fetal circulation, so the P-gp inducer may become the potential therapeutic target of IUGR. Previous studies in our and other laboratories have suggested that P-gp inducers sodium ferulate and tadalafil can effectively reverse the weight loss of fetal mice caused by prenatal tobacco/alcohol exposure or preeclampsia [63, 64]. Sodium ferulate has been widely used to prevent diseases related to reactive oxygen species, such as cancer, cardiovascular disease, and diabetes [65]. In this study, 50 mg/kg·d sodium ferulate did not significantly change the fetal body and placental weights, indicating that this dose has no apparent toxicity to the placenta and fetal development. However, sodium ferulate can reverse the effects of PCE on the fetal body/placental weight, placental P-gp expression, and the fetal serum/placental corticosterone levels, suggesting that P-gp is the prevention and treatment target of IUGR.

The detection of the related epigenetic markers (e.g., H3K9ac and H3K4ac) and the key signal molecules of P-gp in maternal can be used as potential early warning biomarkers of IUGR. In this study, using detecting the clinical specimens, we found that the p-JNK protein level was increased, and YB-1 and P300 were significantly decreased in the male placenta of the IUGR group, while only the YB-1 expression level in the female placenta of the IUGR group was significantly decreased. Furthermore, the placental p-JNK protein levels in the male newborns were negatively correlated with P-gp protein levels, while placental YB-1 expression levels in both females and males as well as P300 expression levels in the females, were positively correlated with P-gp protein levels. These results suggest that JNK/YB-1/P300 pathway may be the common regulatory pathway for P-gp expression inhibition in IUGR neonates under various adverse environments during pregnancy, among which the expression of YB-1 is the most stable and closely correlated with the fetal weight. To further determine the relationship among the P-gp, its important regulator YB-1, and the other IUGR models, we also tested other IUGR rat models induced by prenatal dexamethasone and ethanol exposure. The results demonstrated that the mRNA and protein expression levels of P-gp and YB-1 in the placentas were inhibited, and the placental abcb1a/b expression levels were positively correlated with the body weights in the female and male fetal rats (Additional file 1: Figure S2, S3). Based on these, we propose that YB-1 can be used as a potential early warning target of placental glucocorticoid barrier opening, the occurrence of IUGR, and susceptibility to multiple diseases.

More and more studies have reported that specific genes and their epigenetic modifications in the placenta can be used as biomarkers of abnormal placental and fetal development under adverse environments during pregnancy and can be released into the maternal blood in the form of exosomes [66–68]. It is known that genes expressed in human peripheral blood cells (PBMCs) could overlap with 80% of genes expressed in other tissues [69]. Peripheral blood mononuclear cells are defined as any blood cell with a round nucleus (i.e., lymphocytes, monocytes, or macrophages), which are biological samples that are easily obtained in clinical practice. The epigenetic modification and expression of some genes in PBMCs are consistent with that of tissues and can be used as a substitute biomarker for tissues and organs [70, 71]. Therefore, the use of maternal blood exosomes or PBMCs for early warning of placental biomarkers has great potential. In this study, based on the important role of P-gp and YB-1 in mediating the IUGR occurrence, we proposed that the detection of the expression of P-gp, YB-1, and related epigenetic markers in maternal blood exosomes or PBMC may become an early warning target of IUGR. Unfortunately, we have not collected enough blood samples from pregnant women for further detection and validation of placental epigenetic biomarkers.

## Conclusions

**Fig. 8:**
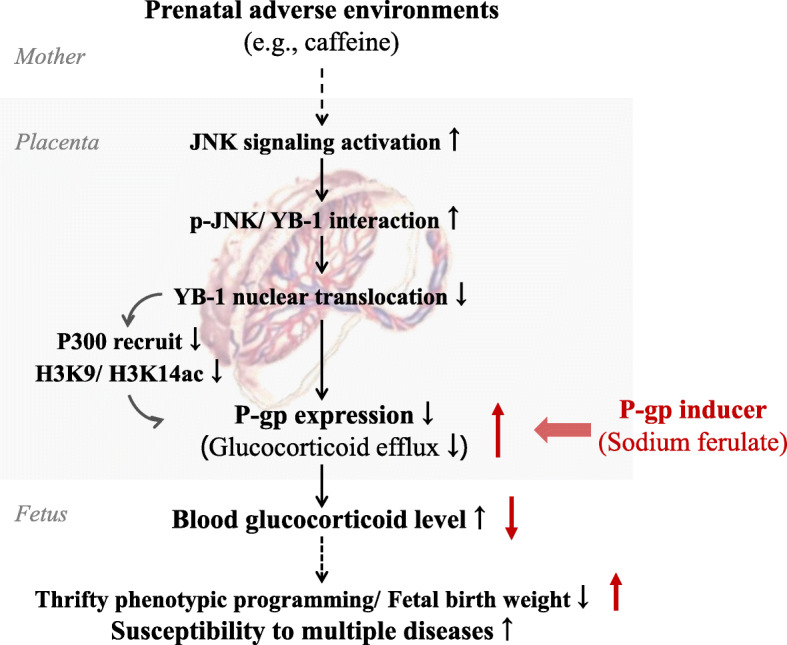
P-glycoprotein (P-gp) expression inhibition mediates placental glucocorticoid barrier opening and fetal birth weight loss. JNK C-Jun N-terminal kinase, YB-1 Y-box protein 1, H3K9/H3K14ac histone 3 Lysine 9/14 acetylation

In conclusion, for the first time, we demonstrate that the inhibition of P-gp expression mediates the opening of the placental glucocorticoid barrier and fetal weight loss in clinical IUGR specimens and PCE-induced IUGR rat model. Further, we used the rat IUGR model and BeWo cells to confirm that the mechanism is related to the activated JNK signal, which in turn inhibits the YB-1 expression and nuclear translocation, thereby directly inhibiting P-gp transcription. YB-1 expression inhibition can also decrease P-gp promoter acetylation and expression by reducing the recruitment of P300 (Fig. 8). Sodium ferulate intervention study confirmed that P-gp inducer can reverse the effect of caffeine on the fetal/placental weights, suggesting that P-gp is a target for the prevention and treatment of IUGR. Finally, the studies of clinical specimens and series IUGR animal models confirmed that the JNK/YB-1 pathway is the common regulation mechanism of P-gp expression, and YB-1 is the potential early warning target of placental glucocorticoid barrier opening, the occurrence of IUGR, and susceptibility of multiple diseases.

## Supplementary Information

**Additional File 1: **Figures S1-S3. **Figure S1.** Schematic representation of the postulated molecular pathway by which inhibits placental P-glycoprotein (P-gp) expression in prenatal caffeine exposure-induced intrauterine growth retardation rat model. **Figure S2.** Changes of fetal body/placental weights, placental P-glycoprotein (P-gp) and Y-box protein 1 (YB-1) expression levels in prenatal dexamethasone exposure (PDE)-related intrauterine growth retardation (IUGR) rat model. **Figure S3.** Changes of fetal body/placental weights, placental P-glycoprotein (P-gp) and Y-box protein 1 (YB-1) expression levels in prenatal ethanol exposure (PEE)-related intrauterine growth retardation (IUGR) rat model.

## Data Availability

The datasets used and/or analyzed during the current study are available from the corresponding author on reasonable request.

## Abbreviations

IUGR: Intrauterine growth retardation
MS: Metabolic syndrome
PCE: Prenatal caffeine exposure
ABCB1: ATP-binding cassette subfamily B member 1
P-gp: P-glycoprotein
RYR: Ryanodine receptor
JNK: C-Jun N-terminal kinase
YB-1: Y-box protein 1
H3K9ac: Histone 3 Lysine 9 acetylation
