# Correction to: P-gp expression inhibition mediates placental glucocorticoid barrier opening and fetal weight loss

**DOI:** 10.1186/s12916-022-02307-2

**Published:** 2022-03-27

**Authors:** Caiyun Ge, Dan Xu, Pengxia Yu, Man Fang, Juanjuan Guo, Dan Xu, Yuan Qiao, Sijia Chen, Yuanzhen Zhang, Hui Wang

**Affiliations:** 1 Department of Obstetrics and Gynaecology, Zhongnan Hospital of Wuhan University, 169 Donghu Road, Wuchang District, Wuhan, 430071 China; 2 Department of Pharmacology, Basic Medical School of Wuhan University, 185 Donghu Road, Wuchang District, Wuhan, 430071 China; 3 Hubei Provincial Key Laboratory of Developmentally Originated Diseases, 185 Donghu Road, Wuchang District, Wuhan, 430071 China

**Correction to: BMC Medicine 19, 1–20 (2021)**

**https://doi.org/10.1186/s12916-021-02173-4**

The production team handling this article mistakenly inverted the order of the corresponding authors. This has since been corrected.

