# Supplementary material for: P-gp expression inhibition mediates placental glucocorticoid barrier opening and fetal weight loss
Source: BMC Med. 2021 Dec 8;19:311. doi: 10.1186/s12916-021-02173-4 (PMC8653610; doi:10.1186/s12916-021-02173-4)
Supplement: Supplementary file 1 — Additional File 1: Figures S1-S3. Figure S1. Schematic representation of the postulated molecular pathway by which inhibits placental P-glycoprotein (P-gp) expression in prenatal caffeine exposure-induced intrauterine growth retardation rat model. Figure S2. Changes of fetal body/placental weights, placental P-glycoprotein (P-gp) and Y-box protein 1 (YB-1) expression levels in prenatal dexamethasone exposure (PDE)-related intrauterine growth retardation (IUGR) rat model. Figure S3. Changes of fetal body/placental weights, placental P-glycoprotein (P-gp) and Y-box protein 1 (YB-1) expression levels in prenatal ethanol exposure (PEE)-related intrauterine growth retardation (IUGR) rat model. [file 12916_2021_2173_MOESM1_ESM.doc]

|  |  |  |  |
| --- | --- | --- | --- |
|  |  |  |  |
|  |  |  |  |
|  |  |  |  |
|  |  |  |  |
|  |  |  |  |
|  |  |  |  |
|  |  |  |  |
|  |  |  |  |
|  |  |  |  |
|  |  |  |  |
|  |  |  |  |
|  |  |  |  |
|  |  |  |  |
|  |  |  |  |
|  |  |  |  |
|  |  |  |  |
|  |  |  |  |
|  |  |  |  |
|  |  |  |  |

|  |  | |  | |
| --- | --- | --- | --- | --- |
|  |  |  |  |
|  |  |  |  |  |
|  |  |  |  |  |
|  |  |  |  |  |
|  |  |  |  |  |

|  |  | |  | |
| --- | --- | --- | --- | --- |
|  |  |  |  |
|  |  |  |  |  |
|  |  |  |  |  |
|  |  |  |  |  |
|  |  |  |  |  |

**Additional file 1**


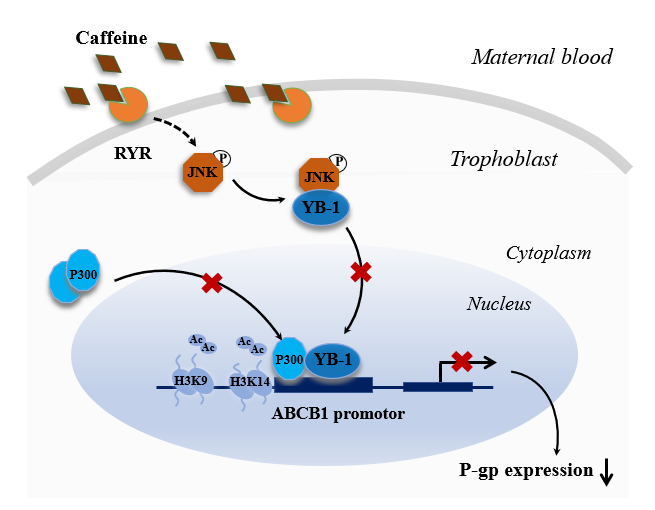


**Figure S1. Schematic representation of the postulated molecular pathway by which inhibits placental P-glycoprotein (P-gp) expression in prenatal caffeine exposure-induced intrauterine growth retardation rat model.** RYR: ryanodine receptor; JNK: C-Jun N-terminal kinase; YB-1: Y-box protein 1; P300: E1A binding protein P300; H3K9/H3K14ac: Histone 3 Lysine 9/14 acetylation.


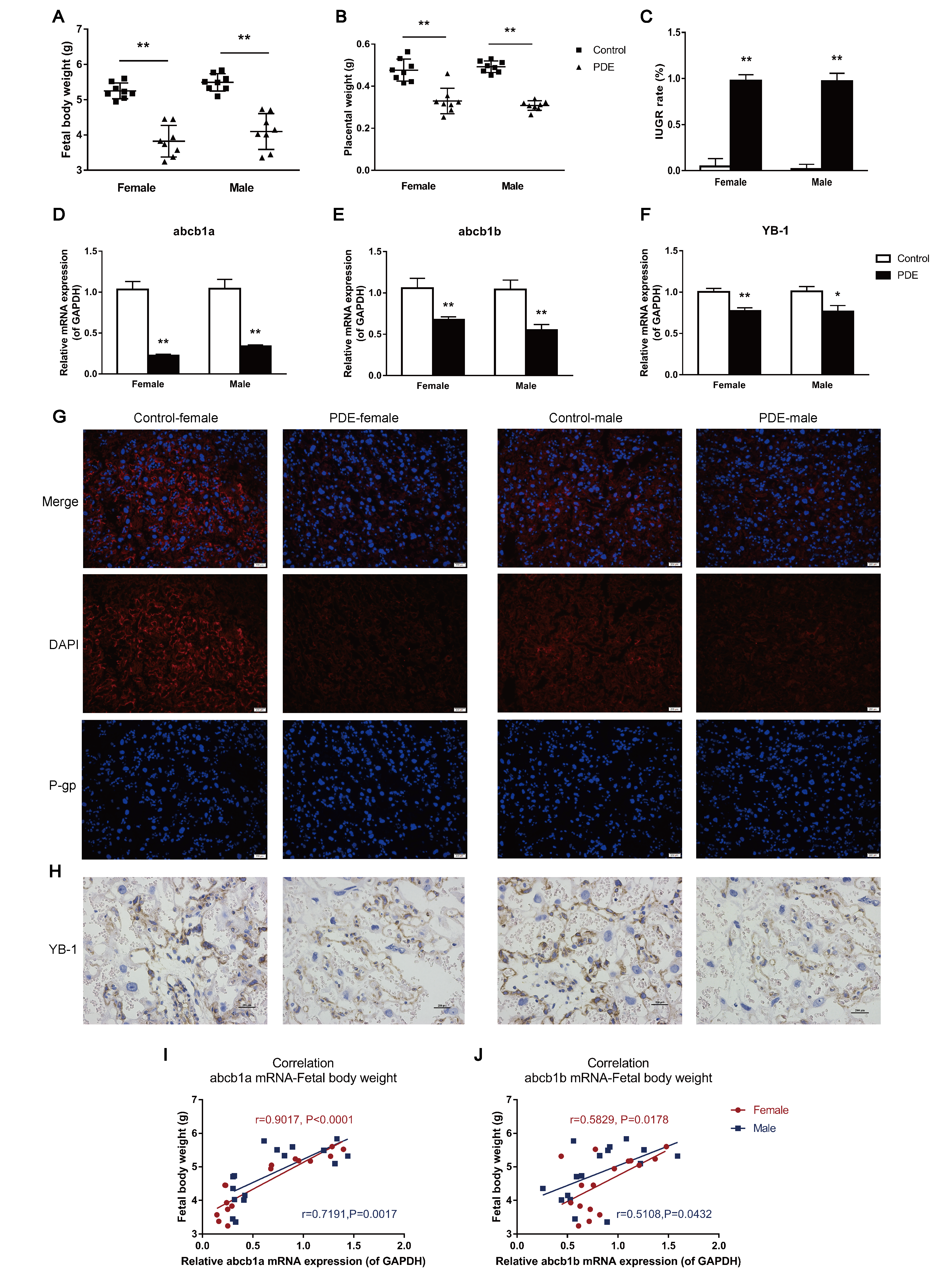


**Figure S2. Changes of fetal body/placental weights, placental P-glycoprotein (P-gp) and Y-box protein 1 (YB-1) expression levels in prenatal dexamethasone exposure (PDE)-related intrauterine growth retardation (IUGR) rat model.** (A) Fetal body weight; (B) Placental weight; (C) IUGR rate; (D-F) Relative mRNA expression levels of ATP-binding cassette, sub-family B, member 1a (abcb1a), abcb1b and YB-1, n=8; (G) Representative photomicrographs of immunofluorescence for placental P-gp (200×); (H) Immunostaining of YB-1 (400×), n=5; (I, J) Correlations between abcb1a or abcb1b mRNA expression levels and fetal body weights, n=16. Mean ± S.E.M. *^*^P*<0.05, ^**^*P*<0.01 *vs.* control.


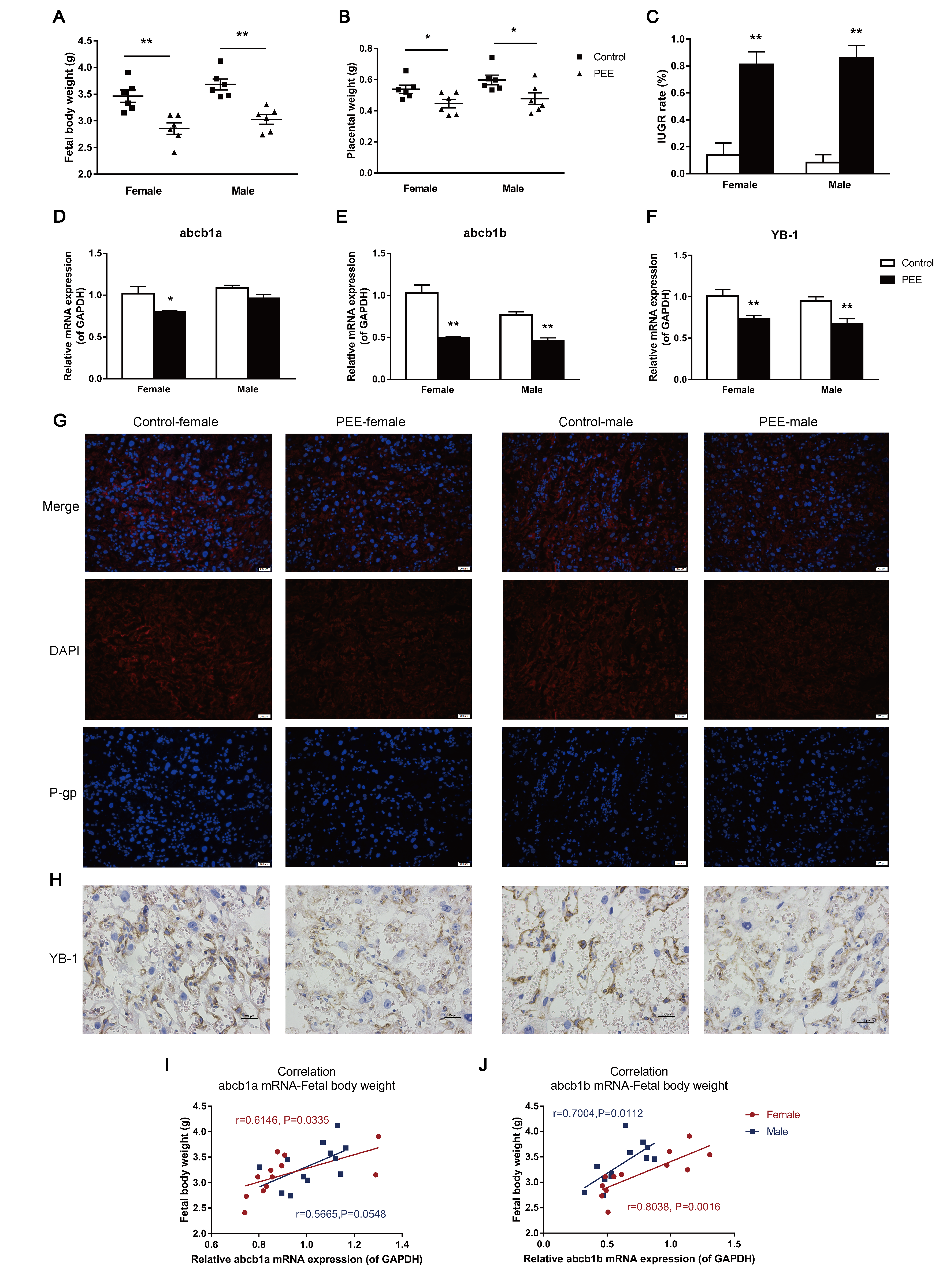


**Figure S3. Changes of fetal body/placental weights, placental P-glycoprotein (P-gp) and Y-box protein 1 (YB-1) expression levels in prenatal ethanol exposure (PEE)-related intrauterine growth retardation (IUGR) rat model.** (A) Fetal body weight; (B) Placental weight; (C) IUGR rate; (D-F) Relative mRNA expression levels of ATP-binding cassette, sub-family B, member 1a (abcb1a), abcb1b and YB-1, n=6; (G) Representative photomicrographs of immunofluorescence for placental P-gp (200×); (H) Immunostaining of YB-1 (400×), n=5; (I, J) Correlations between abcb1a or abcb1b mRNA expression levels and fetal body weights, n=12. Mean ± S.E.M. *^*^P*<0.05, ^**^*P*<0.01 *vs.* control.
